# Feasibility of a Single Pigtail Stent Made by Cutting a Nasobiliary Drainage Tube in Endoscopic Transpapillary Gallbladder Stenting for Acute Cholecystitis

**DOI:** 10.7759/cureus.25072

**Published:** 2022-05-17

**Authors:** Koji Takahashi, Hiroshi Ohyama, Mayu Ouchi, Motoyasu Kan, Hiroki Nagashima, Yotaro Iino, Yuko Kusakabe, Kohichiroh Okitsu, Izumi Ohno, Yuichi Takiguchi, Naoya Kato

**Affiliations:** 1 Gastroenterology, Chiba University, Chiba, JPN; 2 Medical Oncology, Chiba University, Chiba, JPN

**Keywords:** stents, gallbladder, endoscopy, drainage, cholecystitis

## Abstract

Background and objective

In this study, we aimed to evaluate the efficacy and safety of a single pigtail stent made by cutting a nasobiliary drainage tube (NBD stent) by comparing the clinical outcomes of using an NBD stent and those of using a ready-made double pigtail stent (RDP stent) in endoscopic gallbladder stenting (EGBS) for acute cholecystitis.

Materials and methods

This was a single-center retrospective study involving 20 cases that had technical success with EGBS for acute cholecystitis; the patients were divided into two groups: those using NBD stent (NBD group) and those using RDP stent (RDP group). The baseline characteristics and clinical outcomes were compared between the two groups.

Results

There were 13 patients in the NBD group and seven in the RDP group. The rates of clinical success (NBD group: 92% vs. RDP group: 100%, p=0.45) did not differ significantly between the groups. Regarding adverse events, gallbladder perforation occurred in one case in the NBD group; however, no other adverse events occurred in either group (NBD group: 7.7% vs. RDP group: 0%, p=0.45). The stent patency periods did not differ significantly between the groups [NBD group: 43 (12-64) days vs. RDP group: 97 (58-215) days, p=0.17]. The stent patency period in cases of long-term stent placement after EGBS was 1,381 days and 1,579 days in the NBD group and 305 days in the RDP group, respectively.

Conclusion

NBD stents are considered as effective as RDP stents in EGBS for acute cholecystitis. They are highly versatile and can be used for both bridging to surgery and long-term stent placement.

## Introduction

Urgent or early cholecystectomy is the gold standard therapeutic procedure for acute cholecystitis [1]. However, for patients who are likely to undergo surgery, percutaneous transhepatic gallbladder drainage (PTGBD) is considered an alternative treatment. For patients with coagulopathies, those on antibiotics, and those with ascites, PTGBD is not suitable. For such patients, endoscopic transpapillary gallbladder drainage (ETGBD) that does not require needle puncture is a viable option [2]. ETGBD includes endoscopic naso-gallbladder drainage (ENGBD) and endoscopic gallbladder stenting (EGBS). ETGBD is sometimes performed as bridging to surgery (BTS) therapy for acute cholecystitis followed by interval cholecystectomy [3,4]. ENGBD is associated with patient discomfort and the risk of self-removal of the drainage tube. EGBS is considered superior in terms of patient quality of life to ENGBD in that it involves no tube outside the body and there is considerably less discomfort. Specialized stents for EGBS are scarce, and ready-made pigtail plastic stents for biliary stenting are often used for the procedure. However, the distance from the lumen of the gallbladder to the duodenal papilla varies from case to case. And, a longer stent than the ready-made variety is often required. For that reason, the single pigtail stent, which is made by cutting a pigtail nasobiliary drainage (NBD) tube, is often used [5,6]. The advantage of this stent is that its length is adjustable; however, its clinical outcomes compared to the ready-made stent are unclear. In this study, we examined the clinical outcomes of EGBS that uses single pigtail stents made by cutting a pigtail NBD tube (NBD stents) compared to ready-made double pigtail stents (RDP stents) for acute cholecystitis and evaluated the efficacy of such stents.

## Materials and methods

Study design

This was a single-center retrospective study, which included patients who had technical success with EGBS for acute cholecystitis at our hospital between July 2012 and May 2021. Among them, patients with a history of EGBS and patients who underwent PTGBD before EGBS were excluded.

**Figure 1:**
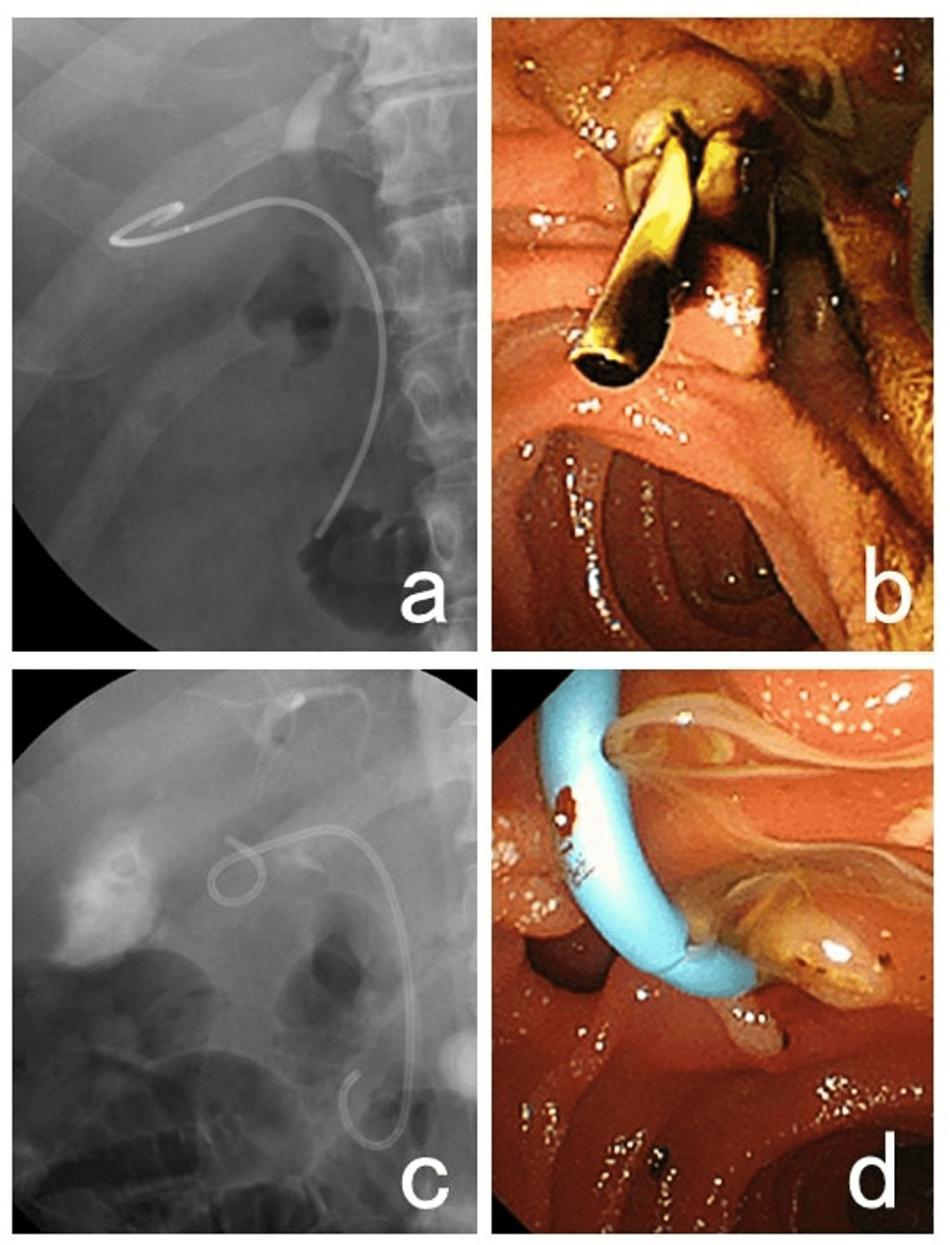
Two types of stents used for endoscopic gallbladder stenting a, b: Stenting using the single pigtail stent made by cutting a nasobiliary drainage tube. c, d: Stenting using the ready-made double pigtail stent

The study participants were divided into two groups; those using NBD stent (NBD group) and those using RDP stent (RDP group) (Figure 1). Baseline characteristics and clinical outcomes were compared between the two groups. The baseline characteristics included age, sex, the status of endoscopic sphincterotomy (EST) performance (classified as follows: not performed, already performed, or performed during the procedure), the duration of the procedure, the diameter of the stent, and the length of the uncurved part of the stent. The clinical outcomes included the clinical success rate, the rate of adverse events, the post-EGBS course, the patency of the stent, and the observation period.

Techniques

**Figure 2:**
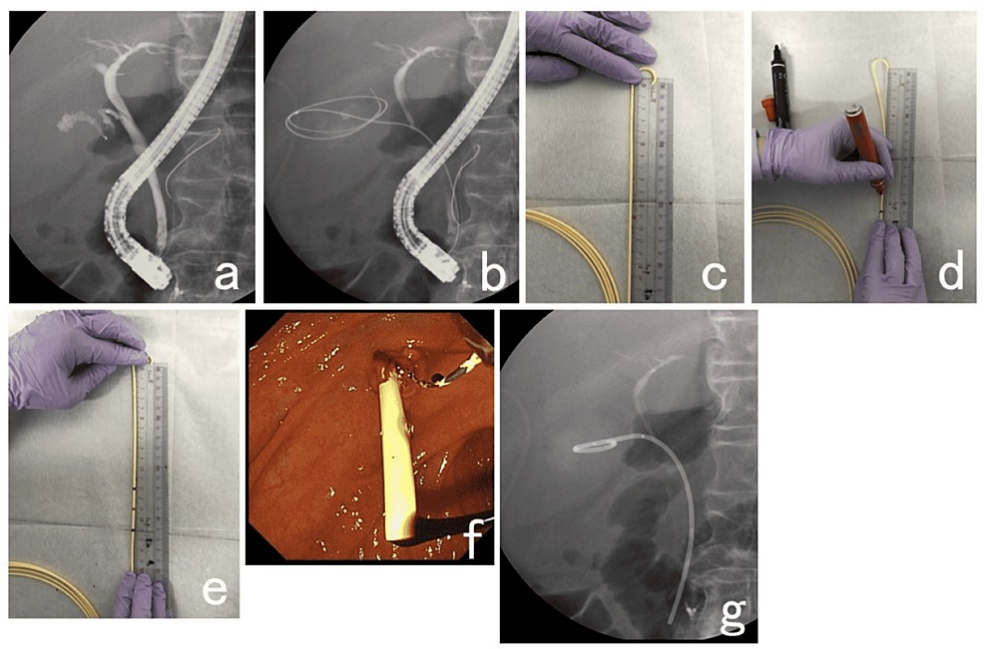
Stenting method of the single pigtail stent made by cutting a nasobiliary drainage tube a: Imaging the gallbladder from the cystic duct with a contrast medium using a catheter. b: Placing a guidewire in the gallbladder. c, d, e: Marking and cutting a required length of the nasobiliary drainage tube and making a stent. f, g: Using the remainder of the nasobiliary drainage tube as the pusher, the pigtail end of the created stent is inserted into the gallbladder, the guidewire is pulled out, and the stent is placed. No side holes were made on the duodenal side of the stent

Before initiating endoscopic treatment, intravenous antibiotics were administered. EGBS was performed using an oblique-viewing endoscope (JF260V, TJF260V, and TJF-Q290V; Olympus, Tokyo, Japan). In the absence of contraindications, carbon dioxide insufflation was used during the procedure. Sodium meglumine amidotrizoate was used as the contrast medium. After imaging the gallbladder from the cystic duct with a contrast medium using an ERCP catheter (PR-104Q-1; Olympus, Tokyo, Japan. MTW; ABIS, Hyogo, Japan. SwingTip; Olympus, Tokyo, Japan), a guidewire (VisiGlide; Olympus, Tokyo, Japan. VisiGlide2; Olympus, Tokyo, Japan. M-Through; ASAHI INTECC, Aichi, Japan. Radifocus; TERUMO, Tokyo, Japan) was placed in the gallbladder. Then, in the NBD stent group, the required length of the pigtail ENBD tube (Daimon; Silux, Saitama, Japan) was measured, marked with a magic marker, and cut. The pigtail end of the newly created stent was coiled into the gallbladder over the guidewire and the distal end was positioned across the major papilla in the duodenal lumen, using the remainder of the naso-biliary tube as the pusher. After inserting the pigtail end of the created stent into the gallbladder, the guidewire was pulled out, and the stent was placed (Figure 2). No side holes were made on the duodenal side. While in the RDP stent group, the ready-made stents (Through & Pass Double Pit; Gadelius Medical, Tokyo, Japan. Zimmon; Cook Medical Japan G.K., Tokyo, Japan. V-System PBD-203; Olympus, Tokyo, Japan) were placed.

Definitions

Clinical success was defined as an improvement in the symptoms of acute cholecystitis after EGBS. The duration of the procedure was defined as the time interval between the insertion of the endoscope and its removal. Adverse events were evaluated according to the lexicon for endoscopic adverse events of the American Society for Gastrointestinal Endoscopy workshop [7]. The patency period of the stent was defined as the duration from stenting to occlusion for stent-occluded cases, from stenting to stent removal for stent-removed cases, and from stenting to the final follow-up appointment for patients with continued stent placement. The baseline characteristics and clinical outcomes were obtained from patients’ medical records.

Written informed consent for EGBS was obtained from all patients. The ethics committee of our hospital approved this study. Consent for the participation of patients in this study was obtained through an opt-out methodology. This study was conducted in accordance with the principles of the Declaration of Helsinki.

Statistical analysis

Data are presented as median with interquartile range or number with percentages. For univariate analysis, the Mann-Whitney U test was used to compare continuous variables, while Pearson’s χ2 test was used to compare categorical variables. All statistical analyses were performed using Bell Curve for Excel (Social Survey Research Information Co., Ltd., Tokyo, Japan).

## Results

Of the 22 patients who were initially included, one with a history of EGBS and one who underwent PTGBD before EGBS were excluded from the study. Finally, 20 patients (14 men and six women) were considered to be eligible for analysis. The ages of our 20 study patients ranged from 49 to 88 years, with a median age of 75 (68-79) years. The number of patients was 13 in the NBD group and seven in the RDP group.

Table 1 shows a comparison of the background characteristics of each group. There were no significant differences in age (p=0.25), the proportion of males (p=0.26), the proportion of cases intended for BTS (p=0.59), the EST status, and the duration of the procedure (p=0.16) between the two groups. As for the diameter of the used stent, the proportion of 7Fr was significantly higher in the RDP group (NBD group: 38% vs. RDP group: 86%, p=0.043), while the length of the uncurved part of the stent was significantly longer in the NBD group (p<0.001).

**Table 1:** Comparison of the background characteristics of the two groups IQR: interquartile range; EST: endoscopic sphincterotomy; EGBS: endoscopic gallbladder stenting

Characteristics	Single pigtail stent made by cutting a nasobiliary drainage tube (n=13)	Ready-made double pigtail stent (n=7)	P-value
Age, years, median (IQR)	70 (68–78)	76 (72–84)	0.25
Sex, male, n (%)	8 (57%)	6 (86%)	0.26
EST status, n (%)			
Not performed	1 (7.7%)	0	0.45
Already performed before EGBS	6 (46%)	4 (57%)	0.64
Performed during EGBS	6 (46%)	3 (43%)	0.89
Procedure time, minutes, median (IQR)	45 (29–73)	28 (26–40)	0.16
Stent diameter, n (%)			
5Fr	3 (23%)	1 (14%)	0.64
6Fr	5 (38%)	0	0.058
7Fr	5 (38%)	6 (86%)	0.043
Length of straight part of stent, cm, median (IQR)	15 (14–17)	10 (10–10)	<0.001

Table 2 shows a comparison of the clinical outcomes in each group. The rates of clinical success (NBD group: 92% vs. RDP group: 100%, p=0.45) and the rate of occurrence of adverse events (NBD group: 7.7% vs. RDP group: 0%, p=0.45) did not differ significantly between the groups. As an adverse event, gallbladder perforation was observed in one case (7.7%) in the NBD group; the concerned patient did not improve clinically after EGBS, and CT imaging revealed a perforation of the gallbladder four days later, necessitating emergency surgery. No other adverse events were observed. In the course of post-EGBS, stent obstruction was seen in two cases, and the stent patency period was three days and 12 days, respectively. In the case with stent obstruction on the third day after EGBS, EGBS was performed again. The other case with stent obstruction on the 12th day after EGBS underwent ENGBD and had an interval cholecystectomy. The proportion of cases that underwent interval cholecystectomy was significantly higher in the NBD stent group (NBD group: 54% vs. RDP group: 0%, p=0.016), and the proportion of cases in which the stent was removed with an endoscope and no cholecystectomy was significantly higher in the RDP stent group (NBD group: 7.7% vs. RDP group: 86%, p<0.001). The stent patency period in cases of long-term stent placement after EGBS was 1,381 days and 1,579 days in the NBD group and 305 days in the RDP group, respectively. There was no significant difference between the stent patency period (p=0.17) and the observation period (p=0.76) between the groups. There were two stent obstructions in the NBD group, but none in the RDP group (p=0.25).

**Table 2:** Comparison of the clinical outcomes in the two groups EGBS: endoscopic gallbladder stenting; BTS: bridging to surgery; IQR: interquartile range

Outcomes	Single pigtail stent made by cutting a nasobiliary drainage tube (n=13)	Ready-made double pigtail stent (n=7)	P-value
Clinical success, n (%)	12 (92%)	7 (100%)	0.45
Adverse events, n (%)	1 (7.7%)	0	0.45
Gallbladder perforation	1 (7.7%)	0	0.45
Post-EGBS course, n (%)			
Stent obstruction	2 (15%)	0	0.25
Interval cholecystectomy	7 (54%)	0	0.016
Long-term placement without removal	2 (15%)	1 (14%)	0.95
Removed during emergency cholecystectomy	1 (7.7%)	0	0.45
Removed with an endoscope and no cholecystectomy	1 (7.7%)	6 (86%)	<0.001
Stent patency period, days, median (IQR)	43 (12–64)	97 (58–215)	0.17
Observation period, days, median (IQR)	273 (28–1,579)	628 (389–1,070)	0.76

## Discussion

In this study, we examined the efficacy of NBD stents in EGBS for acute cholecystitis. Although our study’s sample size was small, there were no significant differences in clinical outcomes between patients who used the NBD stent and those who used the RDP stent. In both study groups, the stent patency was considered sufficient for BTS, that is, the stents could function during the pre-cholecystectomy waiting period. In the NBD group, there were two cases of long-term patency (over 1,300 days). Although it depends on the case, long-term stent placement is considered entirely possible.

Currently, surgical cholecystectomy is the mainstay of the management of acute cholecystitis, depending on the patient's condition and that of each facility. In cases where surgery is either risky or not possible for some reason, the standard approach employed is PTGBD [8]. Cases where PTGBD is unsuitable, such as ascites, abnormal coagulation, and dementia, represent good indications for EGBS [9,10]. Recently, EUS-guided gallbladder drainage (EUS-GBD) has been developed as a new minimally invasive approach. A retrospective study comparing EGBS and EUS-GBD showed that technical success and clinical success rates were significantly higher in the EUS-GBD group compared to the EGBS group; procedure-related adverse event rate was significantly higher in the EGBS group, as was the cholecystitis recurrence rate [11]. EUS-GBD is performed only in a small number of facilities due to its associated high technical difficulty, and it can result in serious complications such as gastrointestinal perforation and the deviation of the stent into the abdominal cavity [12]. Also, in patients taking antithrombotic drugs and those with ascites, the transpapillary approach is more suitable than EUS-GBD.

For EGBS, dedicated stents are scarcely available and not very popular [6]. The distance from the duodenal papilla to the gallbladder varies between individuals. Although it is desirable to have stents of various lengths for EGBS in each facility, it is not realistic due to storage space or cost issues. The advantage of NBD stents in EGBS is that we can avoid stocking different types and sizes of RDP stents and delivery systems. The method of constructing single pigtail stents from NBD tubes is highly versatile because they can be made using an ENBD, a ruler, a magic marker, and a pair of scissors. It can be done in facilities that do not have stents of various lengths. The duodenal side of the stent should be approximately 1 cm from the duodenal papilla to prevent either migration into the common bile duct or duodenal perforation. One disadvantage of the NBD stents is that they cannot be pulled back when inserting the tube. The main concern with single pigtail stents is that duodenal perforation may occur because their duodenal sides are straight; however, in this study, there were no cases of duodenal perforation. There was one case of gallbladder perforation in the NBD stent group, but the causal relationship with the NBD stent is unclear. Further studies involving a large number of cases are required to clarify whether there is a causal relationship.

The single pigtail stent is useful when stent exchange is needed when compared to the double pigtail stent. Straight-shaped duodenal end of the stent enables the easy introduction of the guidewire into the stent. Maintaining the route of the guidewire to the gallbladder is useful for the success of the procedure. EGBS is clinically effective; however, technical success rates are lower than PTGBD or EUS-GBD. Since there are cases where ENGBD is possible even if EGBS is deemed not viable, it is difficult to retrospectively determine whether EGBS and not ENGBD was intended from the beginning of the procedure. For that reason, the technical success rate has not been examined in this study. Further prospective studies are needed to compare the technical success rates of using the NBD stent and those of using the RDP stent in EGBS.

Although it takes time to produce stents (marking and cutting NBD tubes), there was no significant difference in the treatment time between these and ready-made stents in this study. This is due to the fact that guidewire intubation of the gallbladder via the cystic duct is the most crucial and time-consuming step in EGBS and fashioning the NBD stent does not require that much time. There were a few cases of long-term stent patency of 1,000 days or more, and the mechanistic theories behind cholecystitis prevention include the prevention of stone-mediated occlusion of the cystic duct and the drainage of bile via cystic duct around the in-dwelling stent due to the wick effect. NBD stents are considered safe and effective for endoscopic transpapillary gallbladder stenting for acute cholecystitis.

This study has several limitations. Firstly, the number of subjects in our study was small. The small sample size in this retrospective study prevented reaching any robust conclusions about the comparison of the NBD stent and RDP stent. Studies involving a larger number of patients are needed for the further evaluation of the effectiveness and adverse events. Second, this was a retrospective study and was strongly influenced by endoscopists' preferences regarding the indications for EGBS and the selection of stents. Especially, more than half the patients in the NBD group underwent cholecystectomy as compared to none in the RDP group. This raises the possibility of patients in both groups being different enough to adversely impact the conclusions of the study. Finally, even though there were various types of RDP stents, they were examined together as one group.

## Conclusions

NBD stents are considered as effective and safe as RDP stents in EGBS for acute cholecystitis. They are highly versatile and can be used for both BTS and long-term stent placement.
